# Biomechanical determinants of ground contact time and jump height during drop jumps in male athletes

**DOI:** 10.7717/peerj.20947

**Published:** 2026-03-17

**Authors:** Hirotomo Kubota, Kohdai Kishi, Taiyo Kurita, Yohei Takai

**Affiliations:** National Institute of Fitness and Sports in Kanoya, Kanoya, Kagoshima, Japan

**Keywords:** Stretch-shortening cycle, Ground reaction force, Three-dimension motion analysis, Multivariate analysis

## Abstract

**Background:**

Drop jump (DJ) performance is underpinned by a complex interplay of biomechanical factors occurring during the eccentric and concentric phases. This study aimed to identify the biomechanical determinants of DJ performance using partial least squares (PLS) regression analysis.

**Methods:**

Forty-three male collegiate athletes (mean age, height, and mass = 20.1 ± 1.3 years, 1.74 ± 0.07 m, 79.0 ± 23.4 kg) performed DJs from a 0.3-m box while three-dimensional kinematics and ground reaction forces (GRFs) were recorded. Ground contact time, jump height, and reactive strength index (RSI) were analyzed as outcome measures.

**Results:**

PLS regression models demonstrated strong explanatory power for each outcome (*R*^2^*Y*: 92–93%, *p* < 0.001). Ground contact time was negatively influenced by vertical GRFs during the eccentric phase, concentric center of mass (COM) displacement, hip abduction/adduction angle at initial ground contact, and hip and knee joint range of motion (ROM) in the sagittal planes (variable importance in projection (VIP) = 1.30–1.60, weight = −0.23 to −0.19). Jump height was positively influenced by COM displacement and work, vertical impulse and GRF, hip and knee flexion/extension ROMs during the concentric phase and knee angle at initial ground contact (VIP = 1.05–2.68, weight = 0.17–0.41). RSI was positively influenced by vertical GRF during ground contact, vertical stiffness, hip abduction/adduction ROM, concentric COM work and impulse during the concentric phase (VIP = 1.01–2.18, weight = 0.17–0.38).

**Conclusions:**

These findings indicate that larger ROM and greater concentric impulse contribute to jump height but at the cost of longer ground contact, whereas shorter ground contact is promoted by high eccentric vertical ground reaction force and near-neutral hip posture, which can trade off against height.

## Introduction

The drop jump (DJ), a stretch-shortening cycle task, is widely used as a plyometric exercise in sports to assess and enhance explosive lower-limb performance (Hammami et al., 2024). Jump height, ground contact time, and the reactive strength index (RSI) are commonly used as indicators of DJ performance. In fact, high DJ performance has been shown to be associated with superior athletic activities such as straight-line sprinting and change-of-direction maneuvers (Barr & Nolte, 2011; Douglas et al., 2020; Šarabon & Bishop, 2023). As jump height and ground contact time are the components that define RSI and are influenced by the kinetic and kinematic characteristics of the DJ, understanding the biomechanical determinants of DJ performance is essential for optimizing training programs in athletes.

DJ performance is underpinned by a complex interplay of biomechanical factors occurring during the eccentric (descending, braking) phase, the transition from the eccentric to the concentric phase, and the concentric (ascending, propulsion) phase (Bobbert et al., 1986; Hook et al., 2024; Kipp et al., 2018). During the eccentric phase, higher peak vertical ground reaction forces (GRFs) have been shown to induce shorter ground contact time (Douglas et al., 2018). The shift from the eccentric to the concentric phase, which is the brief transition between eccentric loading and concentric output, is critical for efficient energy transfer (Komi, 1984). Individuals who exhibit a peak vertical GRF at the moment the center of mass (COM) velocity reaches zero, corresponding to the transition into the concentric phase, have superior DJ performance (Hook et al., 2024). In the concentric phase, peak GRF and power output are key contributors to achieving greater jump height and improved performance outcomes (Bobbert et al., 1986; Peng et al., 2024). Moreover, vertical stiffness calculated by dividing peak vertical GRF by the vertical displacement of the COM during the eccentric phase acts as key moderators across phases, contributing to energy storage and recoil efficiency (Arampatzis et al., 2001; Kipp et al., 2018). From the viewpoint of kinematics, increased lower-limb joint range of motion (ROM), particularly greater knee flexion, typically results in longer ground contact time due to a deeper countermovement (Bobbert et al., 1986). However, when appropriately increased, ROM can augment joint work and net vertical impulse, thereby contributing to greater jump height (Moran & Wallace, 2007). As noted above, no previous study has comprehensively examined movement phases alongside kinetic and kinematic variables and, in a phase-specific manner, explored which variables contribute to DJ performance. Therefore, it remains unclear which variables in which phase most strongly determine key outcomes such as ground contact time and jump height when kinetics and kinematics are considered together.

Conventional statistical approaches such as multiple regression and principal component analysis (PCA) have been widely used to explore biomechanical determinants of DJ performance, but are often limited by short when confronted with high-dimensional, multicollinear datasets typical of biomechanical research (Hook et al., 2024; Yoshida et al., 2024). Principal component regression, for instance, constructs orthogonal components that maximize variance in predictors but may not be optimal for predicting outcome variables. In contrast, partial least squares (PLS) regression directly constructs components that maximize covariance between predictor and response variables, thus enhancing both predictive power and interpretability in contexts where independent variables are numerous and highly correlated (Liu et al., 2022). By taking the response variable into account during the dimensionality reduction process, PLS provides not only a robust estimation framework but also interpretable loading structures that highlight the relative contribution of each predictor to the outcome. Therefore, PLS regression is particularly suited for biomechanical analyses of complex movements such as the DJ, where temporally structured variables from multiple phases (eccentric and concentric phases) may interact to influence jump outcomes. As a consequence, this study aimed to identify key biomechanical determinants that explain variation in DJ performance, with particular emphasis on ground contact time and jump height (Douglas et al., 2018; Hook et al., 2024; Kipp et al., 2018). Given the multidimensional nature of biomechanical data and potential multicollinearity, we employed PLS regression as a significant approach to extract interpretable and phase-specific predictors for each performance outcome.

This study tested the hypothesis that ROM in the lower-limb joints during the concentric phase would be a determinant of jump height, while vertical GRF and stiffness would be key factors influencing ground contact time. Additionally, it was hypothesized that greater ROM in the hip, knee and ankle joints contributed to a longer ground contact time. Understanding the kinetic and kinematic factors that independently determine ground contact and jump height, two primary components of DJ performance, has practical implications for performance enhancement. Clarifying these determinants can inform the development of posture optimization strategies, instructional cues, and targeted training interventions aimed at improving DJ performance in athletes.

## Materials & Methods

### Study design

This study employed a cross-sectional design. Data were collected between October 2024 and January 2025. All participants completed a single laboratory session lasting approximately 1.5 h. Following a standardized warm-up, participants performed DJs as the primary experimental task. Ground reaction forces and the coordinates of body segments and anatomical landmarks were recorded during the trials.

### Participants

Forty-three male collegiate athletes enrolled in a college of physical education and actively competing in sports (20 baseball players, 10 basketball players, 11 soccer players, and two track and field athletes) were recruited as participants. Mean age was 20.1 ± 1.3 years, mean height was 1.74 ± 0.07 m, and mean weight 79.0 ± 23.4 kg. All participants had over 2 years of competitive experience and underwent sport-specific training 5–6 times per week, with each session lasting 1.5–2 h. According to the athlete taxonomy classification of McKay et al. (2022), the study participants were categorized as Tier 3. None reported any illness and none were taking prescribed medications for cardiovascular, metabolic, or orthopedic disorders during the study period. Participants were instructed to consume their usual meals prior to testing. Additionally, before the session, we confirmed that no participants experienced muscle soreness or fatigue resulting from training on the previous day.

The study protocol was approved by the Ethics Committee of the National Institute of Fitness and Sports in Kanoya (No. 24-1-34). All procedures were conducted in accordance with the Declaration of Helsinki, and written informed consent was obtained from all participants after they were fully informed of the purpose and potential risks.

### Experimental procedures

Prior to the test session, all participants completed standardized dynamic warm-up exercises, including 5 min of running at 10 km/h on a treadmill (mercury, h/p/cosmos, Nussdorf-Traunstein, Germany), approximately 5 min of static and dynamic stretching, and three practice DJs to familiarize themselves with the test. Following at least 10 min of rest after the warm-up session, all participants performed three DJs from a box height of 0.3 m onto force plates. They were instructed to keep their hands on their hips and to jump as high as possible while minimizing ground contact time. A rest period of 1 min was provided between trials. Participants were instructed to refrain from unusually strenuous exercise and excessive food intake on the day before testing. All measurements were conducted during daytime hours, prior to their scheduled sport activities on the test day.

### Data collection (three-dimensional motion analysis and ground reaction forces)

Retro-reflective markers for motion capture (*n* = 47; diameter, 12 mm) were attached to the following anatomical locations of each participant (Miyazaki & Fujii, 2022): third metacarpal heads, radial and ulnar styloid processes, medial and lateral humeral epicondyles, anterior and posterior aspects of the humeral greater tuberosities, acromia, suprasternal notch, xiphoid process, seventh cervical and tenth thoracic vertebra, lower end of the ribs, vertex, bilateral tragi; anterior-superior and posterior-superior iliac spines; greater trochanters, medial and lateral femoral epicondyles, medial and lateral malleoli; calcanei, first and fifth metatarsal heads, and the apex of the second distal phalanges. The three-dimensional marker trajectories were recorded at a sampling rate of 250 Hz using a 16-camera motion capture system (Motion Analysis Corp., Santa Rosa, CA, USA). Horizontal and vertical GRFs were measured at a sampling rate of 1,000 Hz using two force plates (two 9286BA; Kistler, Winterthur, Switzerland) during ground contact in the DJ. Participants were instructed to land with each foot on a separate force plate. The obtained force signals were integrated into the software used to collect the motion analysis system data *via* an analog-to-digital converter.

### Data analysis

We used MATLAB R2024b (MathWorks Inc., Natick, MA, USA) for data analysis. Prior to data analysis, the three-dimensional marker trajectories and the force signals were smoothed using a fourth-order zero-lag Butterworth low-pass digital filter with a cut-off frequency of 20 Hz determined by residual analysis (Winter, 2009).

First, the instants of ground contact and take-off during the DJ were identified based on the vertical GRF (threshold, 20 N) (Harper et al., 2022). Ground contact time was defined as the interval between ground contact and takeoff, and flight time was defined as the interval between takeoff and landing. Next, the eccentric and concentric phases of the DJ were derived from the time series of COM (Hook et al., 2024). The eccentric phase was defined as the period from ground contact to the minimum vertical position of the COM, and the concentric phase as the period from this minimum to take-off.

After calculating the COM velocity as the derivative of the vertical COM position, jump height was estimated using the following: (COM velocity at take-off)^2^/(2 ×g). Reactive strength index (RSI) was defined as jump height divided by ground contact time. Impulse was calculated by integrating the vertical GRF over time during both the eccentric and concentric phases. The ratio of vertical impulse during the eccentric phase to that during the concentric phase was determined, with a lower ratio indicating relatively greater concentric impulse, suggesting more effective utilization of the energy absorbed during the eccentric phase (Winter, 2009).

We modeled the whole body as a rigid-linked skeletal model, incorporating 15 body segments and 14 joints. Body segment inertial parameters for each segment and the whole-body COM were calculated using anthropometric data and scaling equations (Dumas, Chèze & Verriest, 2007). The joint centers were defined in accordance with previous studies (Reed, Manary & Schneider, 1999; Sado et al., 2024). A right-handed local segment coordinate system (SCS) for each segment was defined in each frame (Sado, Yoshioka & Fukashiro, 2017; Sado, Yoshioka & Fukashiro, 2020). Lower limb joint angles were calculated as Cardan angles of the distal SCS relative to the proximal SCS. Joint angles at the hip, knee, and ankle were defined using the following rotation sequences: flexion/extension, abduction/adduction, and internal/external rotation for the hip, flexion/extension, valgus/varus, and internal/external rotation for the knee; and flexion/extension, inversion/eversion and abduction/adduction for the ankle. The ranges of motion (ROMs) were calculated at the hip, knee, and ankle joints to assess the kinematics of the lower extremities and COM during both the eccentric and concentric phases. COM power was calculated as the product of vertical GRF and the vertical velocity of COM.

### Statistical analysis

Data are expressed as means and standard deviations (SDs). We approximated the PLS model by multiple regression using the number of latent variables (LVs) as predictors (Mevik & Wehrens, 2007). Assuming an anticipated model effect size of *f*^2^ = 0.43 (Cohen, 1988), *α* = 0.05, power = 0.80, and four predictors (four LVs), *a priori* power analysis in G*Power (F tests; linear multiple regression *R*^2^ deviation from zero) indicated a required sample size of *N* = 33 (Faul et al., 2009). To ensure PLS stability, we also targeted N≥10 × 4 LVs (*i.e.,* *N* ≥ 40) (Hair et al., 2017). Therefore, the number of participants in this study was greater than 40. The final number of LVs was confirmed *via* 10-fold cross-validation described below (Stone, 1974; Wold, Sjöström & Eriksson, 2001). PLS regression analysis was performed to examine the biomechanical factors contributing to ground contact time, jump height and RSI during the drop jump. Each of the three indices was used as a dependent variable in separate models, with the remaining 44 biomechanical variables serving as predictors.

Prior to the PLS analysis, all explanatory variables were standardized using z-score transformation to ensure comparability in scale (Wold, Sjöström & Eriksson, 2001). To address multicollinearity, Pearson correlation coefficients were calculated among the standardized variables. When the absolute value of the correlation coefficient between two variables exceeded 0.95, one of the two was excluded to avoid redundancy. In such cases, the variable with the larger overall correlation with other predictors was removed, thereby retaining the variable that contributed more uniquely to the variance in the dataset. Following correlation-based filtering, variance inflation factor (VIF) values were computed to further assess multicollinearity, and variables with VIF values exceeding 10 were excluded (Vatcheva et al., 2016).

The optimal number of components in the PLS regression model was determined using 10-fold cross-validation (Stone, 1974). The dataset was divided into 10 equal subsets; nine subsets were used to train the model, and the remaining subset was used to evaluate prediction error. This process was repeated 10 times, each time with a different subset used for validation. The number of components that minimized the root mean square error of cross-validation (RMSECV) was selected as the optimal model. RMSECV served as an indicator of the model’s predictive performance, with lower values indicating better generalizability and reduced risk of overfitting. This metric reflects how well the model can predict unseen data and is commonly used to balance model complexity with prediction accuracy in PLS regression.

To assess the relative contribution of each predictor to the independent variables, variable importance in projection (VIP) scores were computed. A threshold of VIP >1 was used to identify variables with a significant influence on the outcome (Akarachantachote, Chadcham & Saithanu, 2014). In addition, regression coefficients (weights) were examined to determine the direction and strength of associations. Model performance was evaluated based on the coefficient of determination (R^2^Y), cross-validated explained variance (Q^2^), and RMSECV (Liengaard et al., 2021). The assumptions of linear modeling (linearity, homoscedasticity of residuals) were verified using diagnostic plots. All statistical analyses were performed using MATLAB R2024b with the Statistics and Machine Learning Toolbox. The significance level was set at *p* < 0.05.

**Table 1:** Descriptive data on the measured variables.

	Means		SDs	Max	Min
CT, s	0.22	±	0.05	0.38	0.16
Ht, m	0.33	±	0.06	0.51	0.25
RSI, m/s	1.6	±	0.4	2.7	0.95
Stiffness, N/m/kg	397.4	±	131.9	663.7	153.0
RFD, N/s/kg	1,068.5	±	343.8	2,019.2	642.1
Synchro, %	2.3	±	2.5	8.7	0.0
IMP_ECC/CON_	1.0	±	0.1	1.3	0.78
*F*_zero-vel_, N/kg	51.1	±	11.2	74.4	27.6
**Hip joint angle at the moment of ground contact, deg**					
Ext/Flex	−24.9	±	7.6	−10.3	−42.1
Ext/Int rotation	1.9	±	5.2	10.8	−8.8
Abd/Add	−6.2	±	3.2	0.1	−13.3
**Knee joint angle at the moment of ground contact, deg**					
Ext/Flex	−20.6	±	6.0	−9.3	−32.2
Ext/Int rotation	−9.0	±	7.8	4.8	−26.3
Valgus/Varus	3.4	±	3.9	11.5	−5.0
**Ankle joint angle at the moment of ground contact, deg**					
Pla/Dor flexion	0.6	±	2.7	7.3	−7.7
Abd/Add	1.3	±	4.5	10.1	−9.1
Eve/Inv	8.6	±	5.9	24.0	−1.0
**Temporary and GRF variables during the eccentric phase**					
CT, s	0.10	±	0.02	0.17	0.07
IMP, Ns/kg	2.4	±	0.2	2.7	2.1
Mean GRF, N/kg	35.6	±	4.9	47.2	24.8
Peak GRF, N/kg	59.0	±	11.0	82.8	38.8
**Whole body COM**					
Disp, m	−2.05	±	0.50	−0.92	−2.93
Work, J/kg	−4.77	±	0.56	−3.71	−5.89
**Joint work, J/kg**					
Hip	−0.05	±	0.25	0.55	−0.60
Knee	−1.25	±	0.35	−0.45	−2.04
Ankle	1.20	±	0.36	2.15	0.42
**Hip range of motion, deg**					
Ext/Flex	8.6	±	3.8	20.0	2.8
Abd/Add	4.3	±	2.0	8.7	0.7
Ext/Int rotation	8.5	±	2.5	13.3	3.7
**Knee range of motion, deg**					
Ext/Flex	27.9	±	7.2	41.4	15.0
Valgus/Varus	4.4	±	1.7	8.3	2.0
Ext/Int rotation	7.6	±	2.7	13.6	3.0
**Ankle range of motion, deg**					
Pla/Dor flexion	38.0	±	7.2	50.3	18.7
Eve/Inv	6.9	±	3.1	17.8	2.0
Abd/Add	7.2	±	2.6	14.1	2.2
**Temporary and GRF variables during the concentric phase**					
CT, s	0.12	±	0.03	0.21	0.09
IMP, Ns/kg	2.4	±	0.2	3.0	2.0
Mean GRF, N/kg	30.0	±	4.1	38.1	21.9
Peak GRF, N/kg	51.7	±	11.0	74.4	28.0
**Whole body COM**					
Disp, m	2.05	±	0.35	2.80	1.32
Work, J/kg	5.15	±	0.82	7.22	3.64
**Joint work, J/kg**					
Hip	0.00	±	0.38	0.85	−0.85
Knee	−2.05	±	0.50	−0.92	−2.93
Ankle	2.05	±	0.35	2.80	1.32
**Hip range of motion, deg**					
Ext/Flex	26.0	±	8.0	51.4	14.3
Abd/Add	5.4	±	2.1	9.5	1.8
Ext/Int rotation	13.0	±	3.9	24.1	4.8
**Knee range of motion, deg**					
Ext/Flex	48.7	±	7.3	68.0	36.5
Valgus/Varus	7.5	±	3.9	19.4	2.5
Ext/Int rotation	9.7	±	3.0	16.5	4.1
**Ankle range of motion, deg**					
Pla/Dor flexion	54.4	±	4.7	62.9	43.2
Eve/Inv	11.4	±	5.4	31.4	4.6
Abd/Add	12.6	±	3.2	21.7	5.8

**Notes.**

Min and Max, minimum and maximum values among the participants; CT, ground contact time; Ht, jump height; RSI, reactive strength index; GRF, ground reaction force; COM, center of mass; Disp, displacement; Stiffness, vertical stiffness; IMP, impulse; RFD, rate of force development; IMP_ECC/CON_, ratio of impulse during the concentric phase to that during the eccentric phase; *F*_zero-vel_, vertical ground reaction force at the moment of zero velocity of center of mass; Synchro, synchronicity; Ext/Flex, extension/flexion; Abd/Add, abduction/adduction; Ext/Int rotation, external/internal rotation; Pla/Dor flexion, plantar/dorsi flexion; Eve/Inv, eversion/inversion.

**Figure 1:**
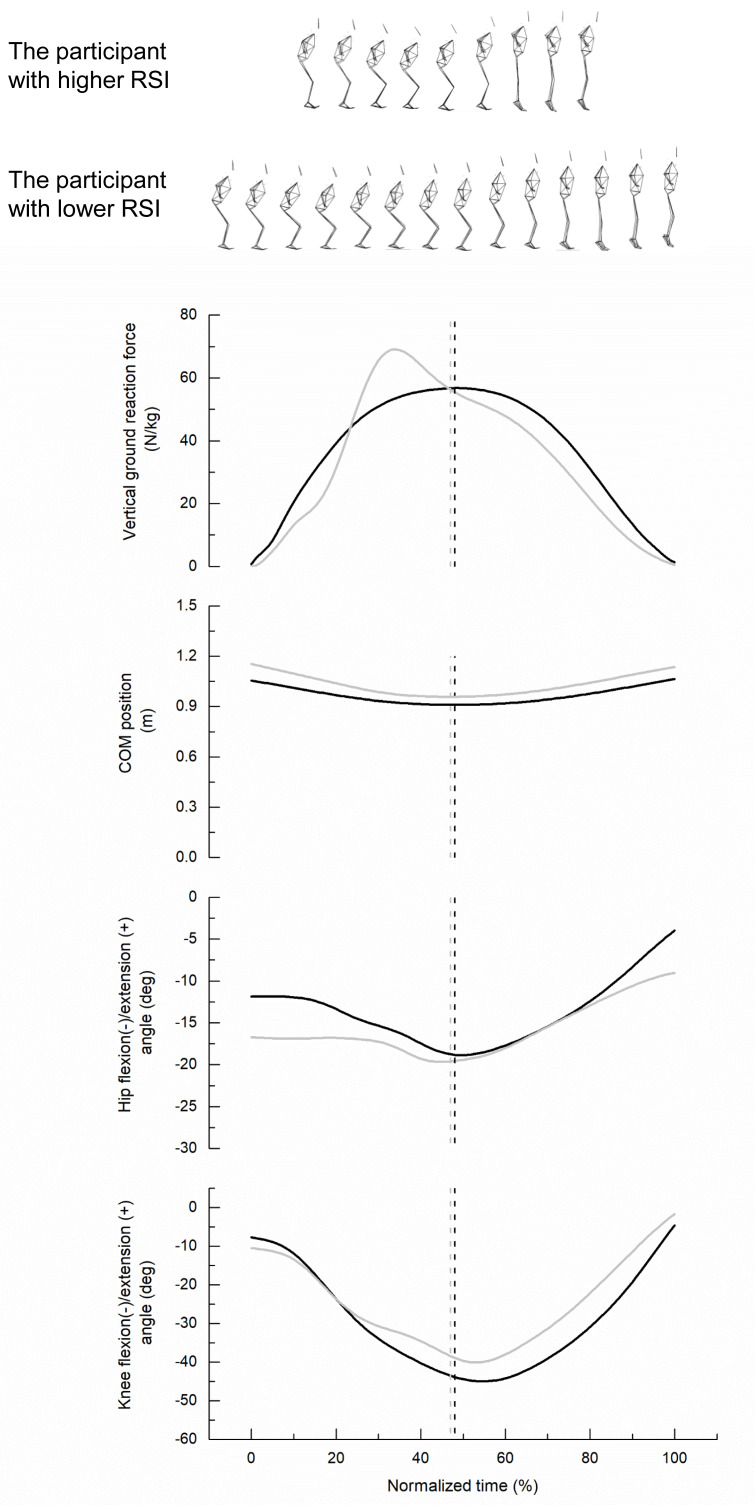
Representative data for vertical ground reaction force, center of mass (COM) position, and hip and knee flexion/extension angles in participants with high and low reactive strength index (RSI). The high-RSI participant corresponds to the 75th percentile, and the low-RSI participant to the 25th percentile of the overall sample. Solid black and gray lines represent the high- and low-RSI participants, respectively. Dashed vertical lines indicate the lowest COM position during ground contact for each participant (black, high RSI; gray, low RSI).

## Results

Table 1 presents the descriptive data of the measured variables. The mean value of DJ performance was 0.22 ± 0.05 s for ground contact time, 0.33  ± 0.06 m for jump height, and 1.57 ± 0.40 m/s for RSI, respectively. Figure 1 illustrates a typical example of vertical GRFs, COM, and hip and knee joint flexion/extension angles for two participants whose RSI value was at the 75th and 25th percentile of the sample. Compared to those with low RSI, participants with high RSI exhibited greater vertical GRFs, smaller vertical COM displacement, and reduced angular changes in hip and knee flexion-extension.

### Factors explaining ground contact time in drop jump

Table 2 summarizes the results of the PLS regression analysis for ground contact time. The model demonstrated strong explanatory power using only the first principal component, yielding an R^2^Y of 93.2%, a Q^2^ of 0.90, and an RMSECV of 0.31 (*p* < 0.001). Independent variables contributing to the first principal component included COM displacement during the concentric phase, mean and peak vertical GRFs during the eccentric phase, hip and knee extension/flexion ROM during the concentric phase, and the angle of hip joint abduction/adduction at initial ground contact. All contributing variables had VIP scores exceeding the threshold of 1. The angle of hip joint abduction/adduction at initial ground contact (VIP = 1.30, weight = −0.19) and vertical GRFs (VIP = 1.58–1.60, weight = −0.23) were significantly and negatively associated with ground contact time, whereas COM displacement (VIP = 1.68, weight = 0.24), hip extension/flexion ROM (VIP = 1.56, weight = 0.23) and knee extension/flexion ROM (VIP = 1.46, weight = 0.21) were significantly and positively associated.

**Table 2:** Summary of partial least squares regression analysis for ground contact time. For each variable, variable importance in projection (VIP) and regression weight (Weight) are reported. Model level metrics include the coefficient of determination (R^2^Y), cross-validated predictive relevance (Q^2^; 10-fold CV), and the root mean square error of cross-validation (RMSECV). VIP≥1.0 is highlighted as an importance threshold.

Explainable variables	Component	VIP	Weight	*R*^2^Y	*Q* ^2^	RMSECV	*p* value
Disp (CON)^*^	1	1.68	0.24	93.24	0.90	0.31	0.000
Mean GRF (ECC)^*^	1	1.60	−0.23				
Peak GRF (ECC)^*^	1	1.58	−0.23				
Hip Ext/Flex (CON)^*^	1	1.56	0.23				
Knee Ext/Flex (CON)^*^	1	1.46	0.21				
Hip Abd/Add@GC^*^	1	1.30	−0.19				
Mean GRF (CON)^*^	2	1.56	−0.25	96.72	0.92	0.28	0.000
COM work (CON)^*^	2	1.01	−0.15				
Ankle joint work (ECC)	2	0.86	−0.27				
IMP (CON)	2	0.41	−0.28				
Ankle Eve/Inv (ECC)	2	0.37	−0.19				
IMP_CON/ECC_	2	0.29	0.20				
Knee Ext/Flex (ECC)^*^	3	1.29	−0.23	98.06	0.94	0.24	0.000
Knee joint work (ECC)^*^	3	1.22	0.33				
Knee joint work (CON)	3	0.98	−0.22				
Ankle joint work (CON)	3	0.82	0.24				
Ankle Eve/Inv (CON)	3	0.64	−0.24				
Knee Ext/Flex@GC	3	0.55	−0.27				
Hip joint work (ECC)	3	0.53	0.26				
Knee Ext/Int rotation@GC	3	0.25	−0.20				
Knee Ext/Int rotation (CON)	3	0.24	0.19				
Disp (ECC)^*^	4	1.58	0.25	98.87	0.96	0.21	0.000
Knee Valgus/Varus (ECC)^*^	4	1.09	−0.33				
COM work (ECC)	4	0.97	−0.18				
Hip Ext/Flex@GC	4	0.92	0.27				
Hip Abd/Add (ECC)	4	0.58	−0.23				
IMP (ECC)	4	0.47	0.11				
CT (ECC)	5	1.88	0.38	99.19	0.96	0.20	0.000
Knee Valgus/Varus@GC^*^	5	1.18	−0.31				
Hip Abd/Add (CON)	5	0.30	0.20				
RFD	6	0.55	0.27	99.41	0.97	0.18	0.000
Hip Ext/Int rotation (ECC)	6	0.39	−0.39				
Ankle Eve/Inv@GC	6	0.17	−0.28				
Hip Ext/Flex (ECC)	7	1.55	−0.24	99.59	0.97	0.17	0.000
Ankle Abd/Add (ECC)	7	0.54	0.31				
Ankle Pla/Dor flexion@GC	7	0.39	0.24				
Ankle Abd/Add (CON)	8	0.64	0.25	99.69	0.97	0.17	0.000
Hip joint work (CON)	8	0.57	0.22				
Knee Ext/Int rotation (ECC)	9	0.89	−0.27	99.77	0.97	0.16	0.000
Hip Ext/Int rotation (CON)	9	0.80	0.22				
Synchro	9	0.67	−0.29				
Ankle Abd/Add@GC	9	0.26	−0.24				
BH	9	0.23	0.28				
Ankle Pla/Dor flexion (CON)	9	0.18	−0.15				
CT (CON)^*^	10	1.88	0.34	99.83	0.97	0.16	0.000
Stiffness^*^	10	1.55	0.26				
Knee Valgus/Varus (CON)^*^	10	1.45	−0.22				
BM	10	0.34	−0.23				
Hip Ext/Int rotation@GC	10	0.23	0.21				
Ankle Pla/Dor flexion (ECC)	10	0.18	0.18				

**Notes.**

* VIP > 1.

CON, the concentric phase; ECC, the eccentric phase; @GC, at initial ground contact; CT, ground contact time; Ht, jump height; RSI, reactive strength index; GRF, ground reaction force; COM, center of mass; Disp, displacement; Stiffness, vertical stiffness; IMP, impulse; RFD, rate of force development; IMP_CON/ECC_, ratio of impulse during the concentric phase to that during the eccentric phase; *F*_zero−vel_, vertical ground reaction force at the moment of zero velocity of center of mass; Synchro, synchronicity; Ext/Flex, extension/flexion; Abd/Add, abduction/adduction; Ext/Int rotation, external/internal rotation; Pla/Dor flexion, plantar/dorsi flexion; Eve/Inv, eversion/inversion.

### Factors explaining jump height in drop jump

Table 3 summarizes the results of the PLS regression analysis for jump height. The model demonstrated strong explanatory power using three principal components derived from 25 independent variables, yielding an R^2^Y of 91.8%, a Q^2^ of 0.81, and an RMSECV of 0.43 (*p* < 0.001). The following seven variables with VIP scores ≥1 contributed to the first principal component: hip and knee flexion/extension ROMs, COM displacement and COM work during the concentric phase, and ankle joint work during the eccentric phase. Concentric-phase vertical impulse and impulse ratio also contributed to this component. Although eight variables were selected in the second principal component, mean vertical GRF and ankle joint work during the concentric phase had a VIP score greater than 1. The third principal component was characterized primarily by COM work and hip joint work during the eccentric phase, and knee joint angle at initial ground contact. Among the variables with VIP scores ≥1, impulse ratio (VIP = 1.90, weight = −0.29), COM work (VIP = 1.23, weight = −0.33) and hip joint work (VIP = 1.16, weight = −0.21) during the eccentric phase were significantly and negatively associated with jump height, whereas all other contributing variables were significantly and positively associated (VIP = 1.05–2.68, weight = 0.17–0.41).

**Table 3:** Summary of partial least squares regression analysis for jump height. For each variable, variable importance in projection (VIP) and regression weight (Weight) are reported. Model level metrics include the coefficient of determination (R^2^Y), cross-validated predictive relevance (Q^2^; 10-fold CV), and the root mean square error of cross-validation (RMSECV). VIP ≥ 1.0 is highlighted as an importance threshold.

Explainable variables	Component	VIP	Weight	*R*^2^Y	*Q* ^2^	RMSECV	*p* value
IMP (CON)^*^	1	2.68	0.41	70.46	0.61	0.63	0.000
COM work (CON)^*^	1	2.43	0.38				
IMP_CON/ECC_^*^	1	1.90	−0.29				
Ankle joint work (ECC)^*^	1	1.47	0.24				
Hip Ext/Flex (CON)^*^	1	1.28	0.21				
Disp (CON)^*^	1	1.16	0.19				
Knee Ext/Flex (CON)^*^	1	1.05	0.17				
Hip joint work (CON)	1	0.94	0.15				
Disp (ECC)	1	0.74	0.11				
Mean GRF (CON)^*^	2	1.48	0.37	85.94	0.78	0.46	0.000
Ankle joint work (CON)^*^	2	1.38	0.27				
Mean GRF (ECC)	2	0.80	0.26				
Peak GRF (ECC)	2	0.63	0.21				
Stiffness	2	0.60	0.17				
Knee Valgus/Varus (CON)	2	0.57	−0.15				
Hip Abd/Add@GC	2	0.49	0.17				
Knee joint work (CON)	2	0.42	−0.13				
COM work (ECC)^*^	3	1.23	−0.33	91.80	0.81	0.43	0.000
Hip joint work (ECC)^*^	3	1.16	−0.21				
Knee Ext/Flex@GC^*^	3	1.14	0.22				
IMP (ECC)	3	0.83	0.28				
Ankle Abd/Add@GC	3	0.81	−0.26				
Knee Valgus/Varus@GC	3	0.67	−0.21				
Knee Ext/Int rotation (CON)	3	0.64	−0.33				
Knee Ext/Flex (ECC)	3	0.48	0.14				
Ankle Pla/Dor flexion (CON)^*^	4	1.27	−0.37	93.37	0.81	0.43	0.000
Knee joint work (ECC)	4	0.84	0.21				
Hip Ext/Flex (ECC)	4	0.79	0.19				
Synchro	4	0.50	0.29				
Hip Abd/Add (ECC)	4	0.45	0.24				
Ankle Eve/Inv@GC	4	0.28	0.18				
Hip Ext/Int rotation@GC	5	0.80	−0.20	95.08	0.79	0.46	0.000
Hip Ext/Flex@GC	5	0.64	−0.25				
Ankle Pla/Dor flexion (ECC)	5	0.45	−0.27				
BM^*^	6	1.17	0.58	96.23	0.80	0.45	0.000
Ankle Abd/Add (CON)	6	0.87	0.19				
Ankle Pla/Dor flexion@GC	6	0.32	0.17				
BH	7	0.69	−0.57	97.01	0.82	0.43	0.000
Hip Abd/Add (CON)	8	0.90	−0.37	97.45	0.83	0.41	0.000
Hip Ext/Int rotation (CON)	8	0.81	−0.15				
RFD	8	0.56	−0.21				
Ankle Eve/Inv (CON)	8	0.52	0.31				
Ankle Abd/Add (ECC)	8	0.19	−0.17				
Knee Valgus/Varus (ECC)	9	0.91	0.18	97.70	0.83	0.41	0.000
Hip Ext/Int rotation (ECC)	9	0.86	0.19				
CT (CON)	9	0.75	−0.26				
Knee Ext/Int rotation (ECC)	9	0.74	0.34				
CT (ECC)	9	0.60	−0.20				
Knee Ext/Int rotation@GC	9	0.45	−0.22				
Ankle Eve/Inv (ECC)	9	0.29	−0.26				

**Notes.**

* VIP > 1.

CON, the concentric phase; ECC, the eccentric phase; @GC, at initial ground contact; CT, ground contact time; Ht, jump height; RSI, reactive strength index; GRF, ground reaction force; COM, center of mass; Disp, displacement; Stiffness, vertical stiffness; IMP, impulse; RFD, rate of force development; IMP_CON/ECC_, ratio of impulse during the concentric phase to that during the eccentric phase; *F*_zero−vel_, vertical ground reaction force at the moment of zero velocity of center of mass; Synchro, synchronicity; Ext/Flex, extension/flexion; Abd/Add, abduction/adduction; Ext/Int rotation, external/internal rotation; Pla/Dor flexion, plantar/dorsi flexion; Eve/Inv, eversion/inversion.

### Factors explaining RSI in drop jump

Table 4 summarizes the results of the PLS regression analysis for RSI. The model demonstrated strong explanatory power using two principal components derived from 24 independent variables, yielding an R^2^Y of 91.6%, a Q^2^ of 0.87, and an RMSECV of 0.37 (*p* < 0.001). Twelve variables were selected in the first principal component, with all having VIP scores greater than 1. In the second primary component, three variables had VIP scores exceeding 1: COM work and impulse during the concentric phase, and impulse ratio. Among the variables with VIP scores ≥1, COM displacement (VIP = 1.03, weight = −0.16) and duration (VIP = 1.49, weight = −0.24) during the eccentric phase; hip extension/flexion ROM (VIP = 1.11, weight = −0.17), knee extension/flexion ROM (VIP = 1.19, weight = −0.19), knee external/internal rotation ROM (VIP = 1.09, weight = −0.17) during the eccentric phase; and knee valgus/varus ROM during the concentric phase (VIP = 1.07, weight = −0.17) and impulse ratio (VIP = 1.39, weight = −0.24) were significantly and negatively associated with RSI. All other contributing variables were significantly and positively associated (VIP = 1.01–2.18, weight = 0.16–0.38).

**Table 4:** Summary of partial least squares regression analysis for reactive strength index (RSI). For each variable, variable importance in projection (VIP) and regression weight (Weight) are reported. Model level metrics include the coefficient of determination (R^2^Y), cross-validated predictive relevance (Q^2^; 10-fold CV), and the root mean square error of cross-validation (RMSECV). VIP≥1.0 is highlighted as an importance threshold.

Explainable variables	Component	VIP	Weight	*R*^2^Y	*Q* ^2^	RMSECV	*p* value
Mean GRF (CON)^*^	1	2.18	0.33	74.51	0.69	0.56	0.000
Mean GRF (ECC)^*^	1	1.66	0.26				
CT (ECC)^*^	1	1.49	−0.24				
Peak GRF (ECC)^*^	1	1.47	0.24				
Stiffness^*^	1	1.28	0.21				
Knee Ext/Flex (ECC)^*^	1	1.19	−0.19				
Hip Abd/Add@GC^*^	1	1.17	0.19				
Hip Ext/Flex (ECC)^*^	1	1.11	−0.17				
Knee Ext/Int rotation (ECC)^*^	1	1.09	−0.17				
Knee Valgus/Varus (CON)^*^	1	1.07	−0.17				
Disp (ECC)^*^	1	1.03	−0.16				
Ankle Abd/Add (CON)^*^	1	1.01	0.16				
Hip Ext/Int rotation (ECC)	1	0.92	−0.14				
Ankle Eve/Inv (CON)	1	0.36	−0.05				
IMP (CON)^*^	2	1.83	0.38	91.59	0.87	0.37	0.000
IMP_CON/ECC_^*^	2	1.39	−0.24				
COM work (CON)^*^	2	1.13	0.33				
Knee Ext/Flex (CON)	2	0.80	0.14				
Hip Ext/Flex (CON)	2	0.79	0.17				
Knee joint work (ECC)	2	0.75	−0.13				
Ankle joint work (ECC)	2	0.60	0.19				
Knee Valgus/Varus (ECC)	2	0.58	0.15				
Hip Ext/Int rotation (CON)	2	0.39	0.10				
Hip joint work (CON)	2	0.37	0.12				
Hip joint work (ECC)^*^	3	1.19	−0.23	96.29	0.93	0.27	0.000
Knee Valgus/Varus@GC^*^	3	1.06	−0.20				
Disp (CON)	3	0.91	−0.14				
Ankle Abd/Add@GC	3	0.84	−0.30				
COM work (ECC)	3	0.75	−0.24				
Knee Ext/Flex@GC	3	0.62	0.28				
IMP (ECC)	3	0.58	0.23				
Knee Ext/Int rotation (CON)	3	0.49	−0.28				
BH	3	0.44	−0.18				
Knee Ext/Int rotation@GC	3	0.34	0.22				
Ankle Pla/Dor flexion (CON)	4	0.80	−0.34	97.20	0.93	0.26	0.000
Synchro	4	0.78	0.23				
Hip Ext/Int rotation@GC	4	0.69	−0.22				
Hip Abd/Add (CON)	4	0.63	0.19				
Hip Ext/Flex@GC	4	0.51	−0.13				
Hip Abd/Add (ECC)	4	0.45	0.30				
Ankle Pla/Dor flexion@GC	4	0.35	0.16				
Ankle Abd/Add (ECC)	4	0.32	0.14				
RFD	4	0.28	−0.13				
Ankle Eve/Inv@GC	4	0.21	0.14				
Ht^*^	5	2.14	0.48	97.84	0.93	0.26	0.000
Ankle joint work (CON)^*^	5	1.58	−0.30				
Knee joint work (CON)^*^	5	1.05	0.19				
BM	5	0.75	0.52				
Ankle Pla/Dor flexion (ECC)	5	0.28	−0.25				
Ankle Eve/Inv (ECC)	5	0.24	−0.21				

**Notes.**

* VIP > 1.

CON, the concentric phase; ECC, the eccentric phase; @GC, at initial ground contact; CT, ground contact time; Ht, jump height; RSI, reactive strength index; GRF, ground reaction force; COM, center of mass; Disp, displacement; Stiffness, vertical stiffness; IMP, impulse; RFD, rate of force development; IMP_CON/ECC_, ratio of impulse during the concentric phase to that during the eccentric phase; *F*_zero−vel_, vertical ground reaction force at the moment of zero velocity of center of mass; Synchro, synchronicity; Ext/Flex, extension/flexion; Abd/Add, abduction/adduction; Ext/Int rotation, external/internal rotation; Pla/Dor flexion, plantar/dorsi flexion; Eve/Inv, eversion/inversion.

## Discussion

This study tested the hypotheses that joint excursion during the concentric phase primarily determines jump height, and that vertical GRF and vertical stiffness contribute to ground contact time. Additionally, it was hypothesized that greater lower-limb joint ROM would be associated with prolonged ground contact time. Based on these findings, these hypotheses were supported. Jump height was explained primarily by impulse during the concentric phase, flexion/extension ROM at the three lower-limb joints, and mechanical work of the COM. Ground contact time was associated with shorter duration when greater vertical GRF was produced during the eccentric phase, whereas longer durations were associated with larger COM displacement during the concentric phase and greater hip and knee joint ROM. While the current results support previous findings, the novelty of this study lies in clarifying how the contributing variables relate to different phases of the DJ.

Ground contact time was explained by 93.2% of the variance through the variables selected in the first principal component, which included concentric-phase COM displacement, hip and knee flexion-extension ROM, eccentric-phase mean and peak vertical GRFs, and hip abduction-adduction at initial ground contact. All variables had VIP scores greater than 1, indicating a high level of contribution as determinants of ground contact time. Mean and peak vertical GRF during the eccentric phase were associated with a shorter ground contact time. This supports a previous finding that braking force is negatively related to ground contact time for sprinters and physically active individuals, suggesting that a greater vertical GRF, especially during the eccentric phase, is advantageous for minimizing ground contact time in DJs (Douglas et al., 2018).

Greater hip adduction angle at initial ground contact contributed to a shorter ground contact time. In this study, the hip joint angle at ground contact was distributed from a neutral posture toward adduction (Table 1). In addition, the hip abduction/adduction angle at initial ground contact exhibited a significant moderate positive correlation with peak vertical GRF (*r* = 0.55, *p* < 0.001) and negative correlation with COM displacement during the eccentric phase (*r* =  − 0.65, *p* < 0.001), suggesting that a greater hip adduction angle at initial ground contact was associated with lower vertical stiffness (*r* = 0.61, *p* < 0.001). Previous studies reported that a decrease in leg stiffness may lead to increased ground contact time for decathletes and physically active individuals (Arampatzis et al., 2001; Peng, 2011). Taken together, the current results suggest that minimizing hip adduction at initial ground contact may help prevent declines in vertical stiffness and contribute to shorter ground contact.

In contrast, greater knee and hip joint flexion/extension ROMs during the concentric phase contributed to a longer ground contact time. Greater ranges of motion in the hip and knee joints during the concentric phase may reflect a movement pattern in which the individual rises from a deeper squat position. In handball players, Bobbert et al. (1986) demonstrated that a jumping style characterized by greater joint torques during DJs, which is reflected by increased ROM, requires a longer concentric time. Furthermore, Peng et al. (2024) reported that individuals exhibiting a bimodal vertical GRF pattern displayed pronounced hip and knee flexion movements, which were associated with longer ground contact times in physically active individuals. Taken together, greater ROM during the concentric phase of a DJ may contribute to prolonged ground contact time. Therefore, hip and knee ROMs appear to be key factors associated with the lengthening of ground contact time.

Greater knee valgus/varus ROM during the DJ contributed to prolonged ground contact time. Previous studies have highlighted the influence of frontal plane knee alignment on landing mechanics. Dewald et al. (2025) demonstrated that increased dynamic knee valgus during single-leg landings was moderately associated with significantly longer ground contact times in recreationally active adults. Supporting this, Tamura, Akasaka & Otsudo (2020) reported that valgus landing postures led to greater mechanical work absorption at the knee and reduced contributions from the hip, which may prolong the deceleration phase and extend ground contact time in healthy females. Furthermore, Wong & Chang (2024) found that individuals with dynamic knee varus exhibited greater peak vertical GRF compared to those with valgus or neutral alignment in male collegiate athletes. These findings suggest that deviations from neutral knee alignment in the frontal plane, particularly in the valgus directions, may impair the efficiency of energy absorption during DJs and contribute to prolonged ground contact time, regardless of peak vertical GRFs.

Jump height was explained by 91.9% of the variance using three principal components derived from 50 independent variables. However, only 17 variables with VIP scores ≥1 contributed to these components. Variables that negatively influenced jump height included the impulse ratio, eccentric-phase COM work, hip and knee joint work, and hip joint external/internal rotation. In contrast, variables that positively influenced jump height included concentric-phase COM work, hip and ankle joint work, hip and knee joint flexion/extension ROMs and ankle joint plantar/dorsiflexion and abduction/adduction ROMs; eccentric-phase ankle joint works; and knee joint angle at initial ground contact; as well as vertical impulse and mean vertical GRF during the concentric phase, and impulse ratio. Jump height is mechanically determined by the vertical COM velocity at take-off, which is, in turn, a result of the vertical impulse generated during ground contact. In fact, a strong positive correlation between jump height and vertical impulse is found for recreationally active males (*r* = 0.92–0.93) (Kirby et al., 2011), whereas the impulse ratio was found to be negatively associated with jump height. Because higher efficiency in the conversion of vertical impulse from the eccentric phase to the concentric phase is considered a key factor for jump performance (Bobbert et al., 1986; Komi & Bosco, 1978), an increased value of this ratio likely reflects lower energy conversion efficiency and may negatively affect jump height. Furthermore, a greater amount of COM and hip joint works during the eccentric phase is likely to prolong the duration of this phase, thereby increasing the vertical impulse during the eccentric phase. This, in turn, might contribute to an increase in the impulse ratio.

The PLS regression analysis revealed that two principal components were sufficient to explain over 90% of the variance in RSI. The variables selected in the first principal component contributed primarily to ground contact time, whereas those in the second principal component contributed primarily to jump height. Because RSI is determined by both jump height and ground contact time, the present findings support this relationship. Kipp et al. (2018) emphasized that vertical stiffness and eccentric-phase COM work are positively related to RSI in male collegiate basketball players. Additionally, Hook et al. (2024) showed that, for both male and female athletes, a higher RSI is associated with enhanced concentric kinetics, particularly when peak concentric force is synchronized with the instance of zero COM velocity, thereby emphasizing the critical role of effective force application timing. The current findings align with and extend previous reports regarding the biomechanical determinants of RSI.

The current findings suggest that greater vertical impulse during the concentric phase, along with increased COM work generated by hip and knee extension, may contribute to enhanced jump height. In contrast, shorter ground contact time was associated with higher vertical force production during the eccentric phase. However, the larger hip and knee excursions that increased COM displacement were linked to prolonged contact time. In addition, greater knee flexion at initial ground contact was positively associated with jump height, whereas a more neutral hip abduction/adduction angle at initial ground contact was associated with shorter ground contact time. Collectively, these results imply that optimizing DJ performance requires a squat-like landing posture, characterized by flexed hip and knee joints at initial ground contact. From this posture, athletes should aim to generate high vertical force and rapidly extend the hip and knee joints to minimize ground contact time while achieving maximal jump height. In practice terms, beyond the standard instruction to “jump as high as possible with minimal ground contact time”, attention to knee flexion and hip alignment at initial ground contact may help facilitate superior DJ performance.

There are several limitations of this study. First, there was a lack of data regarding participants’ force-generating capacity of the lower limb. McBride & Nimphius (2020) demonstrated that individuals with lower strength tend to exhibit different joint work distribution strategies during DJs, relying more heavily on the knee joint to absorb impact forces. In contrast, individuals with higher strength can distribute eccentric loads more effectively across the ankle and hip joints. Second, we did not monitor motor unit activation during DJs. The efficiency of elastic energy reutilization is also influenced by neuromuscular mechanisms, such as pre-activation of muscles prior to ground contact and reflex responses during landing (Taube et al., 2012). Thus, the contribution of ankle joint during the eccentric phase involves not only mechanical energy absorption but also precise coordination with optimal muscle activation strategies to maximize subsequent jump performance. Third, this study focused on male athletes from track and field and ball sports. The findings may differ in endurance athletes or in female athletes. Future studies should investigate participants’ strength levels and neuromuscular activation patterns in conjunction with biomechanical variables to provide a more comprehensive understanding of how physiological and biomechanical factors contribute to DJ performance.

## Conclusions

Partial least squares regression analysis revealed that jump height was determined primarily by the knee angle at initial ground contact and by center-of-mass mechanical work from hip and knee extension and vertical impulse during the concentric phase. In contrast, shorter ground contact time was driven by greater vertical force in the eccentric phase and by near neutral hip abduction/adduction alignment at initial ground contact. The current findings identified key variables that should be emphasized to better elucidate the complex mechanisms underlying DJ performance.

##  Supplemental Information

10.7717/peerj.20947/supp-1 Supplemental Information 1 Matlab code for data analysis

10.7717/peerj.20947/supp-2 Supplemental Information 2 Dataset used in this study

## Additional Information and Declarations

### Data Availability

The following information was supplied regarding data availability: The raw data and code are available in the Supplemental Files.
